# Going Beyond the Injury: Regulatory Conditions Contributing to Latina/o Immigrants’ Occupational Psychosocial Stressors

**DOI:** 10.3389/fpubh.2015.00240

**Published:** 2015-10-20

**Authors:** Airín D. Martínez, Abdel Piedramartel, Jacqueline Agnew

**Affiliations:** ^1^School of Transborder Studies, Arizona State University, Tempe, AZ, USA; ^2^CASA de Maryland-Baltimore, Baltimore, MD, USA; ^3^Environmental Health Sciences, Johns Hopkins Bloomberg School of Public Health, Baltimore, MD, USA

**Keywords:** community-based participatory research, emigrants and immigrants Hispanics/Latinos, occupational exposure, stress, psychological, health policy, immigration policy

## Abstract

**Background:**

Utilizing a psychosocial stress approach, we report psychosocial stressors that Latina/o immigrant day laborers in Baltimore report as workplace hazards and the contextual factors that shape these stressors.

**Methods:**

Through a community–academic partnership, we conducted focus groups (*n* = 18) and key informant interviews (*n* = 9) using instruments developed between academics and the community partner to inquire Latina/o immigrants’ jobs, hazard awareness, occupational illnesses and injuries, and reporting behaviors. We conducted a transcript-based thematic analysis.

**Results:**

The psychosocial stressors that Latina/o day laborers report as dangers at work are anxiety beating the deadline and fear from wage theft, sudden termination and immigration enforcement.

**Discussion:**

More attention needs to be given to Latina/o immigrant day laborers’ occupational psychosocial risks. Policies should be made to lower barriers for Latina/o immigrants to report grievances to state agencies.

## Introduction

Sauter and colleagues reported in the National Occupational Research Agenda that few data exist on safety and health outcomes among workers in contract, part-time, and temporary positions (1). In a systematic review, Virtanen and colleagues found that temporary workers suffered higher psychological issues compared to permanent workers (2). More research is needed to link health effects to business practices (3) in precarious employment, particularly since the traditional theoretical models and assumptions in work organization and psychosocial stress research do not include the growing numbers of contractors, temporary staff, and part-time workers (4).

Foreign-born Latino males comprise a significant proportion of day laborers in the United States (US) labor force (5). Day laborers are temporary workers that seek employment for a short period of time, that can range anywhere from a few hours to a few months, from random and often unknown persons. Street corners or storefront parking lots are the typical recruitment sites for day laborers in the US. However, protected sites are being created for day laborers to seek employment securely, such as workers centers. Workers centers are community-based organizations that combine both advocacy and education to support low-wage workers in obtaining jobs that will pay them fairly and promote their labor rights. Latino day laborers should be considered contingent employees because they “are not formally connected to the social and economic resources of the work organization such as belonging, grievance procedures, health insurance benefits, professional development, [and] promotion…” (4).

Various studies report that Latino workers have more stress than Whites or US-born Latinos in low-wage work such as manual labor, construction (6, 7) agriculture (8), poultry processing (9), and warehouse work (10). In some of these studies occupational stress was related to working in unsafe conditions (10), being poor (5, 11), and being an undocumented immigrant (5, 11, 12). Here, we present the occupational psychosocial stressors that low-wage, Latina/o immigrant day laborers face in Baltimore, MD, USA. We utilize a psychosocial stress approach because it emphasizes the study of stress associated with the experiences of racism and discrimination (13, 14). Fear of immigration enforcement, regardless of immigration status, remains a persistent psycho-environmental stressor among Latinos in the US (15–17). There is little evidence, with the exception of Gleeson (18), that delineates the processes in which local immigration enforcement policies intersect with labor conditions to shape occupational health, much less in a new destination, like Baltimore, which has experienced an increase of 106% of Hispanic/Latinos between the 2000 and 2010 decennial census (19). We end by describing the organizational and regulatory conditions contributing to their psychosocial stressors.

## Materials and Methods

Conducting a research project with Latino immigrant day laborers, who may be unauthorized immigrants, requires methods that promote trust between researchers and community members. We used a community-based participatory research (CBPR) method because it strives to reverse health inequalities through research that is of importance to a community and in collaboration with members of the community – from problem identification to the dissemination of results. More importantly, the end result of any CBPR-guided project is to create positive products – whether they are policies, community-based interventions, or the expansion of methodologies to examine minority populations (20, 21).

The staff at the Casa de Maryland-Baltimore Workers Center and John Hopkins student volunteers wanted to conduct a needs assessment to discover if their current programs were addressing the needs of the Workers Center’s new clientele: older adults and women. At the time, their occupational safety and health trainings focused primarily on occupational risks in construction, yet, women and older adults often engaged in childcare, housekeeping and odd jobs. In lieu of limited data existing regarding Latinos’ health or work conditions in Baltimore (5), we decided to conduct an exploratory qualitative study. The aims of the larger CBPR study were to identify: (1) the health needs and safety hazards faced by Latina/o immigrants; and (2) the economic, political, and social barriers that impede the health of Latina/o immigrant workers in Baltimore. We also sought recommendations for occupational health and safety interventions from immigrant workers, labor, and community leaders.

### Data Collection

#### Participants

Upon receiving IRB approval from the Johns Hopkins Bloomberg School of Public Health, we commenced data collection in 2011. Latina/o immigrant day laborers (ages 20–48) were recruited from a pool of clients utilizing Casa de Maryland’s Baltimore Workers Center and their other services such as citizenship classes, tax preparation, and legal counseling. We also conducted nine key informant interviews with leaders in community development, government employees that worked directly with Latinos to enforce the Occupational Safety and Health Act (OSHA 1970) or Federal Labor Standards Act (FLSA), and labor organizers. We recruited potential key informants from the small, existing pool of Spanish-speaking service providers in Baltimore through leads from Casa de Maryland’s staff and snowball referrals from other interviewees. We decided to conduct interviews with the key informants to identify how persons within government and social service agencies approach the occupational health needs of Latinos in Baltimore and if there was a disjuncture between workers and service providers’ perceptions.

#### Measures

An English and Spanish semi-structured interview guide and focus group protocol were developed in several iterations between the academics and staff at Casa de Maryland, Baltimore, MD, USA. Staff at Casa de Maryland-Baltimore, who are native Spanish speakers, ensured that the final focus group protocol and key informant interview guide were translated in accessible language for persons with lower educational attainment and those from different Latin American countries. The focus group protocol and key informant interview guide included open-ended questions that inquired about: (1) the types of jobs Latina/o immigrants do in Baltimore; (2) identification of safety hazards; (3) illness and injury at work; (4) reporting work injuries and grievances; and (5) recommendations. In addition to these areas, the key informant interview guide also inquired key informants’ history of becoming an advocate for Latina/o immigrants’ labor and occupational safety, and their opinions about the political climate toward Latino workers in Baltimore. A brief demographics questionnaire was also administered to all participants.

The two staff members from Casa de Maryland-Baltimore who co-developed the instruments were trained to facilitate focus groups and write field notes. One staff member was also trained to analyze interview and focus group transcripts. The staff members at Casa de Maryland, as members of the Latina/o community in Baltimore, facilitated three of the four focus groups because it was very important that the focus group participants felt comfortable sharing their experiences. The focus groups took place in a conference room at the partnering organization. The lead author was the note taker during the focus groups. It was important to make note of the focus group participants’ interactions such as their disagreements on a specific topic, and their reaction to the each others’ narratives. The focus groups and key informant interviews were digitally recorded and later transcribed verbatim by an outside transcriptionist. Focus groups lasted between 40 and 60 min, while interviews lasted 40–131 min. All participants received a $20 gift card to a US pharmacy and convenience store, after providing consent.

#### Analysis

We conducted a transcript-based analysis (22) as we wanted to use our findings to construct a survey and future research projects. Our analysis team consisted of the authors: Mr. Piedramartel represented the community perspective, Dr. Agnew is an expert in occupational safety and health, and Dr. Martinez led the qualitative data analysis. The analysis of the transcripts was a three-step process. First, we conducted open-coding sessions identifying *a priori* themes in the transcripts. We developed *a priori* themes based on our specific aims and the areas of the focus group protocol and interview guide. *Ad hoc* themes were those that emerged during our analysis sessions and represent results we did not anticipate. The second step was to collapse our exploratory themes into larger themes, with subthemes in each. Although we used the same themes and guiding research question to analyze both focus group and key informant interviews, we constantly compared the key informants’ perspectives to that of the focus group participants.

The third step was to present our themes and their related excerpts to day laborers utilizing Baltimore Workers Center and Casa de Maryland staff not directly involved in the project. Most of the workers involved in the third stage of analysis did not participate in the focus groups, which provided an opportunity for member checking with persons outside of the study and ensured credibility of our findings.

## Results

We conducted four focus groups with four to five participants in each. There was one focus group with both genders, two focus groups with women, and one group with just men, for a total of 18 participants (female = 10, male = 8). The focus group participants represented six Latin American countries (See Table 1). The focus group participants were between the ages of 20–48 (Median = 35.5 years). They also had been living in the US anywhere from 2 to 18 years (Median = 7 years). There was a large discrepancy in education between female and male focus group participants: 81% of the women had <8 years of education, while 50% of men had at least some secondary education (See Table 1).

**Table 1:** **Sample demographics**.

**Focus group participants** total (*n* = 18)		**Key informants** total (*n* = 9)	
**Mean age ± SD, range**	35 ± 7.10, 20–48	**Mean age ± SD, range**	49 ± 11.83, 27–62
Mean years ± SD in US	14.3 ± 4.29		
**Gender**		**Gender**	
Female	10 (56%)	Female	4 (44.4%)
Male	8 (44%)	Male	5 (55.6%)
**Country of origin**		**Country of origin**	
Brazil	1	Colombia	1
Ecuador	1	El Salvador	2
El Salvador	3	Guatemala	1
Guatemala	2	Venezuela	2
Honduras	1	United States	3
Mexico	10 (56%)		
**Education**		**Education**	
None	1	Some high school	1
<8 years	11	High school graduate	1
Some high school	2	Some college	5
High school graduate	4	Completed college or higher	2
**Types of workers**		**Types of leaders**	
Contractor	2	Community	4
Part-time	2	Government	2
Temporary	14	Labor	3

The key informants (*n* = 9) represented leaders from diverse institutions in Maryland, including labor organizing (*n* = 3), government (*n* = 2) and community-based organizations serving immigrants and/or Latinos (*n* = 4) (See Table 1).

Unlike most day laborers, the male participants found their jobs primarily through interpersonal networks and supplemented their earnings by using Casa’s Workers Center. The women found work using a combination of leads from the Workers Center, interpersonal networks, and temporary staffing agencies, which hired them to clean hotel rooms or office buildings. In fact, both female day laborers and the key informants stated that women never sought work in designated day laborer street locations because it was culturally unacceptable and women faced sexual harassment from Latino day laborers.

Here, we report one set of findings that were unexpected and distinct from our *a priori* themes. Although there are demographic differences between the key informants and focus group participants, two key informants had an insider perspective with the focus group participants because they were also immigrants in low-wage, contingent work. Still, the largest chasm between the responses provided by the workers in the focus groups and the key informants were their responses to the following question: “What are potential dangers that Latino immigrants face at work?” The informants immediately initiated their response with physical and environmental hazards present in a variety of occupations, but primarily focusing on construction sites for men and hotel housekeeping for women. The key informants also discussed the physical injuries that occurred in these jobs such as fractures, sprains, lacerations, chemical exposure, eye injuries, and skin irritations. However, the focus group participants went beyond the injury – they identified potential dangers at work that contributed to feeling anxiety, fear, and stress at work. The potential dangers at work that reflect psychosocial stressors for Latina/o immigrant day laborers are: (1) anxiety to beat the deadline; (2) fear of wage theft and sudden termination; and (3) the fear of immigration enforcement at the workplace.

*Anxiety to beat the deadline* is the condition of being pressured by one’s supervisor to complete tasks expeditiously. This was an occupational risk that was reported by men in construction and manual labor, and women in industrial and hotel cleaning and factory work.

*Well, now what they are doing in construction is that they are pressuring you so that you finish your job faster. And one works with tools that are very dangerous. And so, since we go around nailing with pistols, there is always the risk that you do not target it well and you nail your hands…for doing the job faster because of the boss’s pressure, then you pressure yourself, too*.

–Mexican male, Carpenter

*Look, when I worked in the cans, the risk was that if I got nervous that the can would fall on me and it would fall to my feet and they are heavy and they can hurt you… But if one’s nerves betray you, and whatever happens, it does not improve the situation*.

–Guatemalan female, Cleaner/Factory Worker

The workers claimed that the anxiety from performing quickly made them more inclined to physical occupational injuries, such as the examples above. Many workers, not just Latino immigrant day laborers, have pressures to perform quickly in their jobs; however, what is distinct about this group is that they chose not to communicate this distress to their supervisors. They even evaded reporting occupational injury and continued working injured or ill for fear of termination – “Although one hand does not work with the other yes, but you have be useful to work, because if you do not accomplish, no, then they dismiss you” (Mexican female, housekeeper). Losing one’s job is of great concern considering that as predominantly unauthorized immigrants, they do not qualify for federal public assistance or unemployment benefits. What is worse, none of the focus group participants had a regular source of medical care, so they would not only be anxious from working in a frenzied state, but would also not have means to treat their occupational injuries. In addition to being in temporary positions that did not provide employer-based health insurance, there were few Spanish-speaking health care providers in Baltimore, and few clinics offering low-cost indigent care to undocumented patients.

The second psychosocial stressor that the workers reported as a pervasive danger at work was the fear of wage theft and sudden termination. *Fear of wage theft* and *sudden termination* results when workers are not compensated for part or all of their hours worked, or are suddenly fired. Wage theft was prevalent in the Latino immigrant community in Baltimore as focus group participants, labor organizers, and government employees emphasized how problematic it was.

Look what is most normally heard, and the most common, what usually happens is that they [Latino immigrants] don’t get paid. This is not a new problem, an old and huge and permanent problem. It’s what is heard the most… I have advised a few people that… it happened to me. And it didn’t happen to me once, or twice, it happened like three or four times…

Salvadoran male, electrician and immigrant advocate

The key informants and focus group participants all believed that wage theft and sudden termination were forms of retaliation from an employer when a Latina/o immigrant worker reported an unsafe work environment, requested tools or safety equipment, or reported becoming injured or ill at work. The workers themselves reported the uncertainty of being paid was a constant stressor that they faced entering a job. “But only for a day, two days, one week, but many times one goes and they risk that they will not pay one also” (Ecuadorian female). Many Latino immigrants in Baltimore worked overtime, which is over 40 hours a week in the US, in order to finish their assigned tasks, but they were often not properly compensated for that extra time. Despite being undocumented, all persons working in the United States are entitled to workers’ compensation, a minimum wage, and overtime (23). Employers are also obligated to provide a safe work environment, safety training, and equipment that will prevent occupational injury and illness (23). Since the likelihood of finding another job quickly was so uncertain, Latino immigrants were afraid of making any negative statements about their work conditions to their employers or reporting it to the appropriate state agencies.

*“Are you sick? Do not work.” But there are people who say, “Here [in the US], you do not come as a tourist, here you have to work or give the opportunity to another person.” They have said that to me. So, one does not want to lose their job. When one has a little job, you take care of it, and so that you do not lose it, you will risk it and you say, “I have to bear it and make an effort.” And, there one goes*.

Guatemalan female, Industrial Cleaner

What was interesting in the focus group discussions was how participants attributed much of this economic abuse to documented Latino supervisors, who were either authorized immigrants or US-born Latinos. Three key informants, including two who were previously day laborers, verified this condition separately. “It could be a construction company, it could be a landscaping company, or whatever, remodeling, or whatever, almost always they are Hispanics. It is painful to say it,” (Venezuelan female community organizer). It may be the case that the image of a Latina/o supervisor is more salient among these participants because it is more disappointing to experience discrimination from someone they ethnically identify with, but that differed in immigration status. From the focus group discussions, this *Latino-on-Latino abuse* was perceived as one of the most offensive worker injustices that particularly affected recent arrivals.

*And sadly it is our own people that are doing this. I just saw it a month ago, that people, our own people here, abuse, take advantage of the people who have gotten here more recently… Because they think, they think that they are new, that they are with fear, and that they won’t be able to do anything*.

Salvadoran male, electrician, immigrant advocate

One danger of working as a temporary employee is that there are few if any formal contracts about the employment terms and conditions as temporary employees. In the case below, the worker was owed $1,800 from his employer. With the services of Casa de Maryland’s legal team, he was able to win his case and was granted $800 by the court. However, that success was short won.

*Well we sued the person and- and well, he promised to pay in payments. Eh, he didn’t want to pay all at once but he said, “Well, I will pay this much.” But then he began issuing checks without funds, and… at the end of the day we withdrew the case and that’s how it was*.

Guatemalan organizer and restaurant worker

Once an employer is fined for wage theft, there is no collections department that is part of the Division of Labor, Licensing and Regulation (DLLR), the State of Maryland’s labor agency, or government advocate to obtain those monies. These outcomes are very disappointing for a person who has lost time and money, and more importantly, faith in the system meant to be helpful with workplace abuse. Moreover, this demonstrates that the change needed in order for the system to powerfully deter employers from mistreating immigrant workers, particularly day laborers.

The last psychosocial stressor that Latina/o immigrant workers reported was the threat of immigration enforcement at their workplace. If one was working for a large company, there was a greater possibility of becoming a victim of an Immigration Customs Enforcement (ICE) raid and subsequent deportation. A raid is when ICE agents search and seize a workplace for having undocumented workers present or on payroll. There were enough incidents within Baltimore that supported its existence and constant threat, as reported both by the focus group participants and the key informants. During our study, Baltimore’s Police Department was an active participant in Secure Communities, which was a federal program administered by the Department of Homeland Security to enlist local law enforcement to apprehend and then hand over undocumented immigrants with serious criminal offenses to ICE. More undocumented persons have been apprehended for traffic violations than for felonies (24, 25).

We came to learn that many Latina/o immigrants opted to work for smaller employers, temporary agencies, and day jobs to avoid the constant surveillance of ICE.

*But, I think also that the danger that we have is that I know cases of persons that are very scared to work with the companies because immigration is coming to the jobs and taking people out. And, the people are very scared that they will send them to their country and that is the major problem at the moment… I think that is the danger that mainly now is attacking the people*.

Salvadoran female, housekeeper, 31

Nevertheless, working for smaller employers did not free Latino immigrants from their employers’ threats of contacting ICE if they did not comply with unjust work conditions, such as wage theft or mistreatment.

*Then…they don’t talk, they are afraid, they are intimidated, but because they are also intimidated at their jobs… They [supervisors] say, ‘If you don’t do this [or] you start bothering, I’ll call immigration; I’ll do this.’ Things like that*.

(Guatemalan organizer)

The fear of ICE raids or someone reporting them to ICE was especially striking among mothers, as they emphasized the larger implications of their apprehension on their family’s wellbeing. Their main fear was that they would be detained by ICE and there would be no one to care for their children: “…like she says, sometimes they do not even give you a chance to go and advise someone that, that one’s children are in school, to go and pick them up, and then they stay there alone. Then, it is also another danger,” (Mexican female housekeeper). In a separate focus group, an Ecuadoran participant shared that the community never asks of the whereabouts of persons apprehended by ICE. Mothers’ fear of immigration enforcement is shaped from the uncertainty of being detained and not given the opportunity to arrange caregiving and family responsibilities with those that remain.

During our member-checking sessions, the participants revealed that some Latina/o immigrants do not even take public transit, walk through Downtown Baltimore, or seek medical care at emergency departments because they wanted to avoid any contact with local police. The avoidance of public spaces was a common strategy for Latina/o immigrants to avoid contact with local law enforcement who acted as an extension of federal immigration authorities. However, they had to work to meet their and their family’s material needs. The effect of local immigration enforcement in Baltimore impinged Latina/o immigrant day laborers’ ability to seek medical services, report unjust work conditions or work-induced injuries or illnesses to the appropriate regulatory agencies. Latina/o immigrants’ legal status was an additional technique with which employers could circumvent regulations and just practices to their contingent workers in the informal labor market.

## Discussion

Through a psychosocial stress approach, we presented the occupational psychosocial stressors among a category of contingent workers, Latina/o immigrant day laborers, seeking employment in Baltimore’s informal labor economy. The stressors that the participants identified as unique to their positions as unauthorized day laborers were anxiety to beat the deadline and fear of wage theft, sudden termination, and immigration enforcement at the workplace. Although most occupational health interventions for Latina/o immigrants are created to prevent occupational illness, injury and death, attention needs to be placed on their mental health, too. As the participants brought to our attention, their psychosocial conditions at work are also related to their vulnerability of becoming physically injured. These Latina/o day laborers believe that psychosocial stressors are also chronic occupational hazards.

The focus group participants were distinct from most key informants because they went beyond the physical occupational hazards and physical injury to discuss the many contextual factors that shape their workplace stress, including working for contractors and temporary agencies, and not being authorized to work in the US. These day laborers understood that working directly for a company provided benefits, job security, and organizational membership, which circumvent mistreatment from supervisors. However, the fear of surveillance from any government agencies, particularly local law enforcement or immigration, prevented them from working with more legitimate employers. Moreover, regardless of gender or occupation, these workers attributed *both* their need to work for smaller employers and the discrimination from the supervisors to their undocumented status.

Being contingent workers in the informal labor market creates opportunities for contractors and supervisors to economically and psychologically mistreat Latina/o immigrants in Baltimore. Since they are temporary workers, who often do not even fill out an application, they cannot provide sufficient evidence, such as a formal contract or timecard, to report grievances to the state labor agency. If they are undocumented, they often lack state-issued identification cards to enter the government buildings where they would file grievances with the state labor agency. The State of Maryland did not provide driver’s licenses to undocumented immigrants until late 2013. A follow-up study is needed to assess whether the licensing of undocumented Latinos in Baltimore has improved their ability to report worker-related injury and illness and labor rights violations to state agencies.

**Figure 1:**
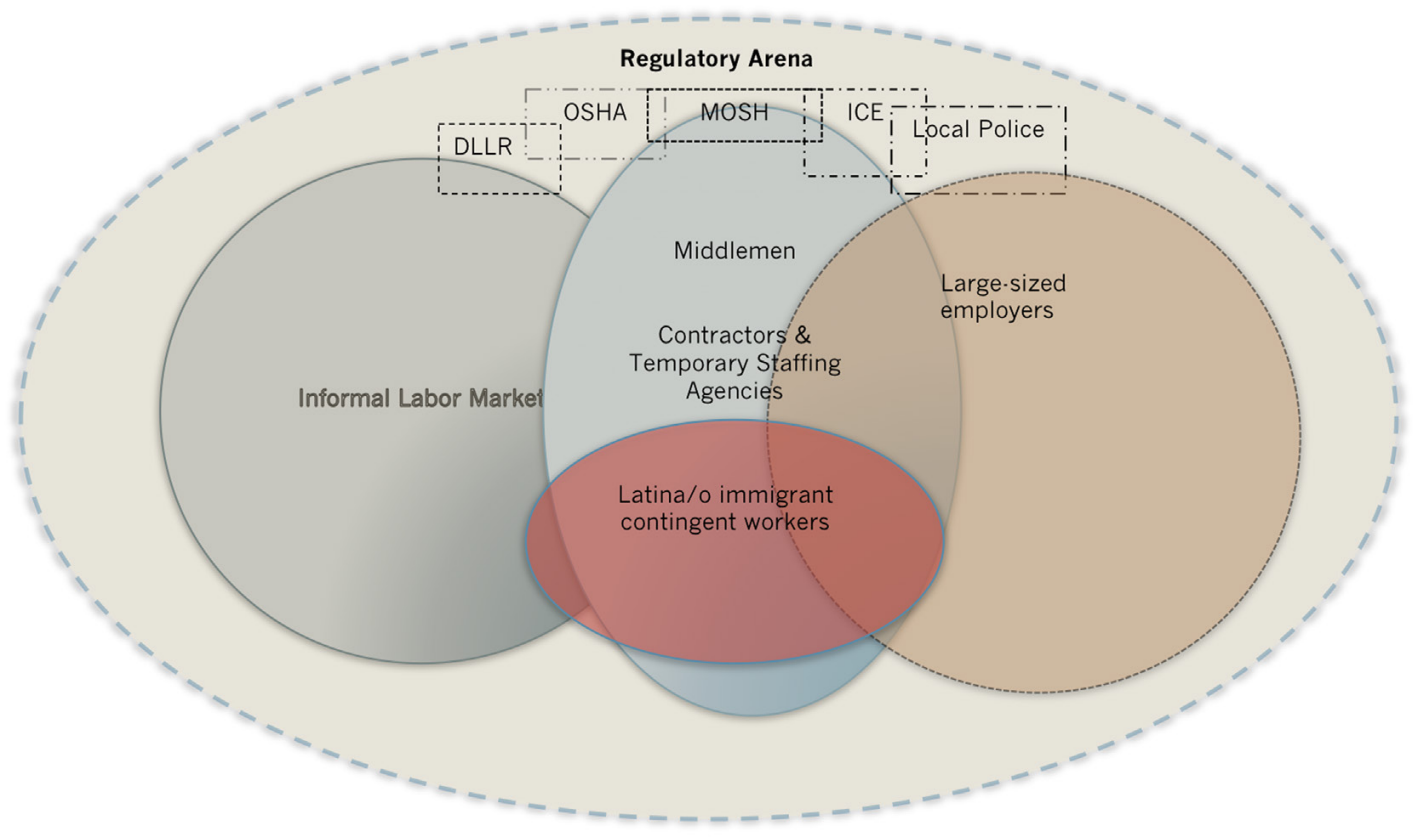
**Diagram of the regulatory arena that shapes Baltimore Latina/o Immigrants’ psychosocial stressors**.

Government agencies do not adequately monitor or sanction smaller employers for adverse treatment toward contingent workers. As one of our key informants shared (a compliance manager for the state’s occupational safety agency) occupational health grievances can only be filed for persons currently employed at the worksite where they are experiencing the issue. Moreover, given that the population of Latino immigrants is fairly recent in Baltimore, during the time of this study there were only a handful of Spanish-speaking employees working in the state labor department and the occupational safety agency that could assist this population. So, temporary and day laborers with finite work assignments in manual labor, service, and hospitality jobs face administrative barriers communicating with the necessary agents and proving their identity and employment. These organizational deficiencies impinge Latino immigrants’ possibility of reprimanding the employer. Combine this oversight with the hyper-policing of Latina/o immigrants in Baltimore due to immigration enforcement policies, and their anxiety and fears at work are intensified (See Figure 1).

Gallagher (4) asserts that the traditional theoretical models and assumptions in work organization and psychosocial stressors research need to include the growing numbers of contractors, temporary staff, and part-time workers. The model in occupational health that has been utilized most to examine the conditions of low-wage laborers is the Demand-Control Model (26). This model proposes that the more control a worker has in their job, the more they can mediate the stress from the demands made at work. However, if a worker in the lower levels of the occupational ladder lack control at their workplace they often cannot buffer high demands on the job, resulting in negative psychological responses such as stress, anxiety, and fear.

The Demand-Control model is helpful in understanding one of the reasons why Latina/o immigrant contingent workers are experiencing these psychosocial stressors – their low-wage, low-occupational status. However, it does not identify *how* the social and political economic environment shapes those demands. Analyzing the responses from a diverse group of Latina/o immigrants in Baltimore, we have identified that during the recent recession and the increase of immigration enforcement that there are a number of demands being made on Latina/o day laborers. Our findings can also expand the Demand-Control Model by discussing some of the organizational and regulatory conditions that limit Latina/o immigrant workers’ control of the situation. Latina/o immigrants’ limited control is not solely a result of being in low-wage and low-occupational status jobs, but also their position in the racialized labor market.

The situation of Latina/o immigrant contingent workers is unique in comparison to other disenfranchised workers because they have an added layer of discrimination at the workplace: their undocumented status. However, undocumented status has been constructed to represent “illegality” and “criminality” through a series of exclusionary and carceral immigration policies the last two decades in the United States (27). These policies have also been targeted toward racially profiling Mexicans and other Latin Americans. Labor exploitation is legitimized because being an undocumented Latina/o is highly criminalized in the US. It is important to analyze the occupational risks of Latina/o day laborers through a psychosocial stress approach to capture the intersection of these vulnerabilities – being in low-wage, high-demand jobs, as non-English speaking racial/ethnic minorities, and undocumented laborers. Moreover, our community partner alluded during our analyses that these exclusionary practices hinder Latina/o immigrants’ adaptation to the US and to be integrated into the community.

Latino immigrants in Baltimore definitely add a new meaning to the notion of “working with stress” when we examine their unique challenges as immigrants in an urban area not accustomed to their linguistic needs or their vulnerability. The creation of anti-immigrant policies has provided more power to employers to exploit Latina/o immigrants in this new destination. Being an undocumented laborer should not justify the threat of wage theft, sudden termination, or immigration apprehension. It promotes a dual labor market and the added stress to Latina/o immigrants who are already in dangerous and stressful jobs like construction and hotel housekeeping.

To conclude, extant research about Latina/o immigrant day laborers has primarily focused on males and Mexican immigrants in agriculture and construction. In this study, we include female workers and persons from diverse Latin American countries. This was an exploratory study with a small sample size and may not be representative of Latinos in Baltimore. After presenting the workers’ recommendations for reducing occupational risks for Latina/o immigrant day laborers, Casa de Maryland’s Workforce Development and Education managers decided to modify and implement a “Safety Backpack” intervention (28) that would fit the needs of both their female and male day laborers.

Unlike other day laborer studies, we report on day laborers that sought work in a variety of locations, including protected sites, such as Casa de Maryland-Baltimore’s Workers Center. Future directions include examining how this diversity affects occupational health outcomes, and a more intense study of female day laborers working for temporary staffing agencies. It would also be important to compare occupational psychosocial risks between day laborers seeking work at protected work sites like workers centers, temporary agencies and social networks, and non-protected work sites like street corners.

## Author Contributions

AM, AP, and JA contributed in the conception and design of the current research project. AP and AM were responsible for the acquisition of the data. AM, AP, and JA all conducted the qualitative data analysis. AM drafted the manuscript. AM, AP, and JA revised the manuscript before submission and provided final approval of the manuscript.

## Conflict of Interest Statement

The authors declare that the research was conducted in the absence of any commercial or financial relationships that could be construed as a potential conflict of interest.
